# Expression of Protease-Activated Receptor 1 and 2 and Anti-Tubulogenic Activity of Protease-Activated Receptor 1 in Human Endothelial Colony-Forming Cells

**DOI:** 10.1371/journal.pone.0109375

**Published:** 2014-10-07

**Authors:** Tiago M. Fortunato, Dina S. Vara, Caroline P. Wheeler-Jones, Giordano Pula

**Affiliations:** 1 Department of Pharmacy and Pharmacology, University of Bath, Bath Spa, United Kingdom; 2 Department of Comparative Biomedical Sciences, Royal Veterinary College, University of London, London, United Kingdom; Johns Hopkins University, United States of America

## Abstract

Endothelial colony-forming cells (ECFCs) are obtained from the culture of human peripheral blood mononuclear cell (hPBMNC) fractions and are characterised by high proliferative and pro-vasculogenic potential, which makes them of great interest for cell therapy. Here, we describe the detection of protease-activated receptor (PAR) 1 and 2 amongst the surface proteins expressed in ECFCs. Both receptors are functionally coupled to extracellular signal-regulated kinase (ERK) 1 and 2, which become activated and phosphorylated in response to selective PAR1- or PAR2-activating peptides. Specific stimulation of PAR1, but not PAR2, significantly inhibits capillary-like tube formation by ECFCs *in vitro*, suggesting that tubulogenesis is negatively regulated by proteases able to stimulate PAR1 (e.g. thrombin). The activation of ERKs is not involved in the regulation of tubulogenesis *in vitro*, as suggested by use of the MEK inhibitor PD98059 and by the fact that PAR2 stimulation activates ERKs without affecting capillary tube formation. Both qPCR and immunoblotting showed a significant downregulation of vascular endothelial growth factor 2 (VEGFR2) in response to PAR1 stimulation. Moreover, the addition of VEGF (50–100 ng/ml) but not basic Fibroblast Growth Factor (FGF) (25–100 ng/ml) rescued tube formation by ECFCs treated with PAR1-activating peptide. Therefore, we propose that reduction of VEGF responsiveness resulting from down-regulation of VEGFR2 is underlying the anti-tubulogenic effect of PAR1 activation. Although the role of PAR2 remains elusive, this study sheds new light on the regulation of the vasculogenic activity of ECFCs and suggests a potential link between adult vasculogenesis and the coagulation cascade.

## Introduction

ECFCs are the most recently identified subtype of endothelial progenitor cells (or EPCs) that originate from unidentified precursors co-isolated with human peripheral blood mononuclear cells (hPBMNCs) [1]. Because of their high proliferative and pro-vasculogenic potential [2], ECFCs are prime candidates for the development of cell therapies aiming to revascularise damaged tissues and stimulate tissue regeneration [3]. There is, therefore, a great deal of interest in understanding the regulatory mechanisms underlying the pro-vasculogenic activity of ECFCs.

Several aspects of the cell physiology of ECFCs remain to be elucidated. In particular, it is of pivotal importance to understand how ECFCs mediate vasculogenesis, which in contrast to angiogenesis is the formation of blood vessels *de novo* (i.e. not by branching from existing vasculature) and plays a critical role in repairing damaged tissues [4]. In common with mature endothelial cells and other subtypes of EPCs, vascular endothelial growth factor (VEGF) appears to play a critical role in stimulating the vasculogenic activity of ECFCs, which is commonly assessed *in vitro* by measuring capillary-like tube formation on Matrigel [5]. In addition to VEGF, several other paracrine factors have been suggested as potential stimulators of the vasculogenic activity of ECFCs, including transforming growth factor β (TGFβ) [6], erythropoietin [7], prostacyclin [8], osteoprotegerin [9] and Dickkopfs 1 (DKK1) [10].

Here, we have investigated the expression and function of PARs in ECFCs. PARs are irreversibly activated by cleavage of their extracellular domain by extracellular proteases, which include thrombin [11], trypsin [12], tryptase [13] and coagulation factors VIIa and Xa [14]. The cleavage by proteases unmasks a ‘peptide agonist’ domain of the extracellular domain of the receptors. When unmasked, the ‘peptide agonist’ domain acts as a tethered ligand, interacting in an intramolecular manner with the extracellular portion of the receptor, which induces receptor activation and its coupling with intracellular signaling pathways [15]. PAR activity is critical for vascular homeostasis and central to coagulation and haemostasis [16].

Previous reports of the expression of a member of the PAR family in different EPC subtypes prompted investigation of the expression of this family of receptors in ECFCs [17]–[19]. Our interest in PAR expression and function in ECFCs derives from the fact that local accumulation of active proteases following stimulation of the coagulation cascade by tissue damage might play a relevant role in the regulation of ECFCs at the site of vascular injury.

In this study, we first identified PAR1 and PAR2 amongst the surface markers expressed by peripheral blood ECFCs. Subsequently, we investigated the effect of PAR1 and/or PAR2 activation on cell signalling and functional responses using selective activating peptides mimicking the tethered ligand sequences [20]. Taken together, we describe a novel PAR1-dependent mechanism of inhibition of ECFC-dependent tubulogenesis.

## Experimental Procedures

### Cell culture

Peripheral blood was obtained by venepuncture from the median cubital vein of healthy drug-free volunteers. Participants were informed about procedure and purpose of blood collection. They expressed their consent in written form. Written consent forms for all participants are kept within the Department of Pharmacy and Pharmacology at the University of Bath and the Local Ethics Committee of the University of Bath has approved the consent procedure and the venepuncture protocol. The cell isolation procedure has been described previously [2]. ECFCs were obtained from the peripheral blood mononuclear cell (PBMNC) fraction of whole human blood, which was separated by density gradient centrifugation method using Histopaque (1.077±0.001 g/ml, Sigma, Poole, UK). PBMNCs were isolated from one donor (i.e. no blood pooling) and seeded at a density of 2×10^5^ cells/cm^2^ on collagen-coated dishes in complete medium (i.e. EBM-2 medium plus EGM-2 Bullet Kit supplements, Lonza, Walkersville, US) containing 12% fetal bovine serum (FBS). Cell culture medium was replaced every 2 days to maintain adequate nutrients levels and remove unattached cells. Colonies appeared between with 14–21 days of culture and were separately expanded. Cell passaging and seeding ahead of experiments was performed by cell detachment using Accutase (Life Technologies, Carlsbad, US). Cells were characterised by FITC-labelled Ulex europaeus agglutinin (UEA) staining, acetylated LDL intake was performed as previously described [21] and immunofluorescence staining for vascular endothelial (VE)-cadherin or von Willebrand Factor (VWF) up to passage 8. Experiments were performed on cells between passages 4 and 6 and were repeated with cells from at least 3 independent isolations (i.e. 3 different donors).

### RT-PCR and qPCR

For RT-PCR, total RNA was extracted from ECFCs and PBMNCs using TRIzol Plus RNA Purification Kit (Life Technologies, Carlsbad, US). The cDNA was obtained using ImProm-II Promega Reverse Transcription System (PROMEGA Corporation, Madison, US) and was selectively amplified by traditional reverse transcriptase polymerase chain reaction (RT-PCR) as previously described [21] (PAR1: 5′-AATCAGGAGGACGTTTGTG and 5′-CTGTGGTGTATCCCATGCAG; PAR2: 5′-TGAAGATGGTCTGCTTCACG and 5′-TCTGCATCTGTCCTCACTGG; PAR3: 5′-AAAAGCATCCACAGGGTCAC and 5′-GAAAGCCCTCATCTTTGCAG; PAR4: 5′-GCACGTA GGCACCATAGAGG and 5′-TGTATGGCTCAGTGCTGCTG; β-Actin 5′-CAATGAGCTGA GAGTAGCCC and 5′-GGGTGTTGAAGGTCTCAAAC). Total RNA prepared with TRI Reagent (Life Technologies, Carlsbad, US) from human lung fibroblasts, human myofibroblasts, ECFCs, human glomerular endothelial cells and epithelial Human Kidney 2 cells (HK2) was donated by Miss Cristina Beltrami and Dr Donald Fraser (Department of Nephrology, University of Cardiff, UK). The cDNA was generated using High Capacity cDNA Reverse Transcription Kit (Life Technologies, Carlsbad, US). qPCR was performed on a ViiA7 Real-Time PCR System (Life Technologies, Carlsbad, US). PAR1, PAR2 and GAPDH reaction products were quantified by Power SYBR Green PCR Master Mix (Life Technologies, Carlsbad, US) with 300 nM gene-specific primers (PAR1: 5′-GTATCCCATGCAGTCCCTCTCC and 5′-GTAATGCGCAATCAGGAGGACG; PAR2: 5′-TCT GCATCTGTCCTCACTGG and 5′-TGAAGATGGTCTGCTTCACG; GAPDH: 5′-AGCCGCATC TTCTTTTGCGT and 5′-TGACGAACATGGGGGCATCA; VEGFR2: 5′CCAGTGTCATTTC CGATCACTTT and 5′-GGCCCAATAATCAGAGTGGCA; VEGFA: 5′-AGGGTCTCGAT TGGATGGCA and 5′-AGGGCAGAATCATCACGAAGT; CXCR4: 5′-CCCACAATGCCAG TTAAGAAGA and 5′-ACTACACCGAGGAAATGGGCT; SDF-1: 5′-ATTCTCAACACT CCAAACTGTGC and 5′-ACTTTAGCTTCGGGTCAATGC; IL8: 5′-AACCCTCTGCACCCAGTTTTC and 5′-ACTG AGAGTGATTGAGAGTGGAC). The amplification of a single PCR product was confirmed by melting curve analysis. Gene-specific mRNA levels were estimated by the 2^−ΔΔCt^ analysis and normalized against GAPDH levels to obtain relative changes in gene expression, as previously described [22].

### PAR stimulation

Selective activation of PAR1 or PAR2 was achieved by treating the cells with synthetic agonist peptides displaying the sequence of the endogenous agonist peptide of the targeted receptor [20] These were used at a final concentration of 50 µM for different incubation times depending on the type of assay. The sequence of the activating peptides is the following: H-Thr-Phe-Leu-Leu-Arg-NH_2_ for PAR1, 2-Furoyl-Leu-Ile-Gly-Arg-Leu-Orn-NH_2_ for PAR2 and 2-Furoyl-Orn-Leu-Arg-Gly-Ile-Leu-NH_2_ for the scrambled control.

### Immunoblotting

ECFCs were lysed in radioimmunoprecipitation assay (RIPA) buffer in the presence of protease and protein phosphatase inhibitors (Sigma, Poole UK). Extracts from platelets and human umbilical vein endothelial cell (HUVECs) were utilized as controls and obtained in a similar manner. Proteins were separated by sodium dodecyl sulfate polyacrylamide gel electrophoresis (SDS-PAGE) and immunolabelled as previously described [23]. Antibodies for PAR1 and PAR2 (N19) were from Santa Cruz Biotechnology (Santa Cruz, US). Phospho- ERK1/2, ERK1/2, CD31 and VEGFR2 antibodies were from Cell Signaling Technology (Danvers, US). The antibodies for integrin IIb and VWF were from Enzo Life Sciences (Farmingdale, US) and Dako (Glostrup, Denmark), respectively. Densitometric analyses were performed using ImageJ 1.46r (Wayne Rasband, National Institute of Health, US).

### Immunofluorescence

ECFCs were grown on cover slips to 60–70% confluence and fixed/permeabilized in ice-cold methanol for 10 min. Following fixation, cells were washed in PBS and blocked with 1% bovine serum albumin (BSA) for 30 min. Subsequently, the slides were incubated with anti-PAR1, anti-PAR2, VE-cadherin (Santa Cruz Biotechnology, Santa Cruz US) or VWF primary antibody (Dako, Glostrup, Denmark) (1∶50) for 1 h at room temperature. After three washing steps, slides were then incubated with Alexa Fluor 488 Rabbit Anti-Mouse and Alexa Fluor 546 Donkey Anti-Goat IgG (1∶200) (Life Technologies, Carlsbad, US) for 1 h and 4′,6-diamidino-2-phenylindole (DAPI, Sigma-Aldrich, Poole UK) (1∶100). Incubation with secondary antibodies and DAPI alone was used as a negative control. Cells were mounted in Vectashield HardSet Mounting Medium (Vector Laboratories, Peterborough UK) and examined on a LSM 510 META confocal microscope (Carl Zeiss AG, Jena, Germany).

### *In vitro* capillary-like tube formation assay

Growth Factor Reduced Matrigel (BD Biosciences, Oxford UK) was utilized to provide extracellular matrix for cell culture. 65 µl of Matrigel were added to each well of a 96-well plate and incubated at 37°C for 30 minutes. 10,000 cells/well were then added in 100 µl of tube formation assay buffer (i.e. EBM-2 medium containing 2% FBS). Cells were cultured at 37°C/5%CO_2_ in the presence of 50 µM PAR1- and/or PAR2-activating peptide and/or 25–100 ng/ml VEGFa_165_ (R&D systems, Minneapolis US) or basic Fibroblast Growth Factor (FGF) (R&D systems, Minneapolis US) with or without pharmacological treatments (50 µM PD98059, Sigma-Aldrich, Poole UK) and phase contrast images were captured 4 hours after treatment using a EVOS FL microscope with an UPlan FL N 4X/0.13 objective. Experiments were repeated a minimum of three times and the vasculogenic response was measured as total number of tubes normalized to control (scrambled peptide) using the Angiogenesis Analyzer plugin of ImageJ (Gilles Carpentier, Faculté des Sciences et Technologie, Université Paris Est, Creteil Val de Marne, France). Validation experiments of this assay using human umbilical vein endothelial cells (HUVECs) are shown in Figure S2.

### Statistical analysis

Data are expressed throughout as mean ± SEM and presented using Prism 5 (GraphPad Software, La Jolla, US). Statistical significance was analyzed by one-way ANOVA with Bonferroni post-test (for parametric data) and Kruskal-Wallis analysis with Dunn’s post-test (for non-parametric data).

## Results

**Figure 1:**
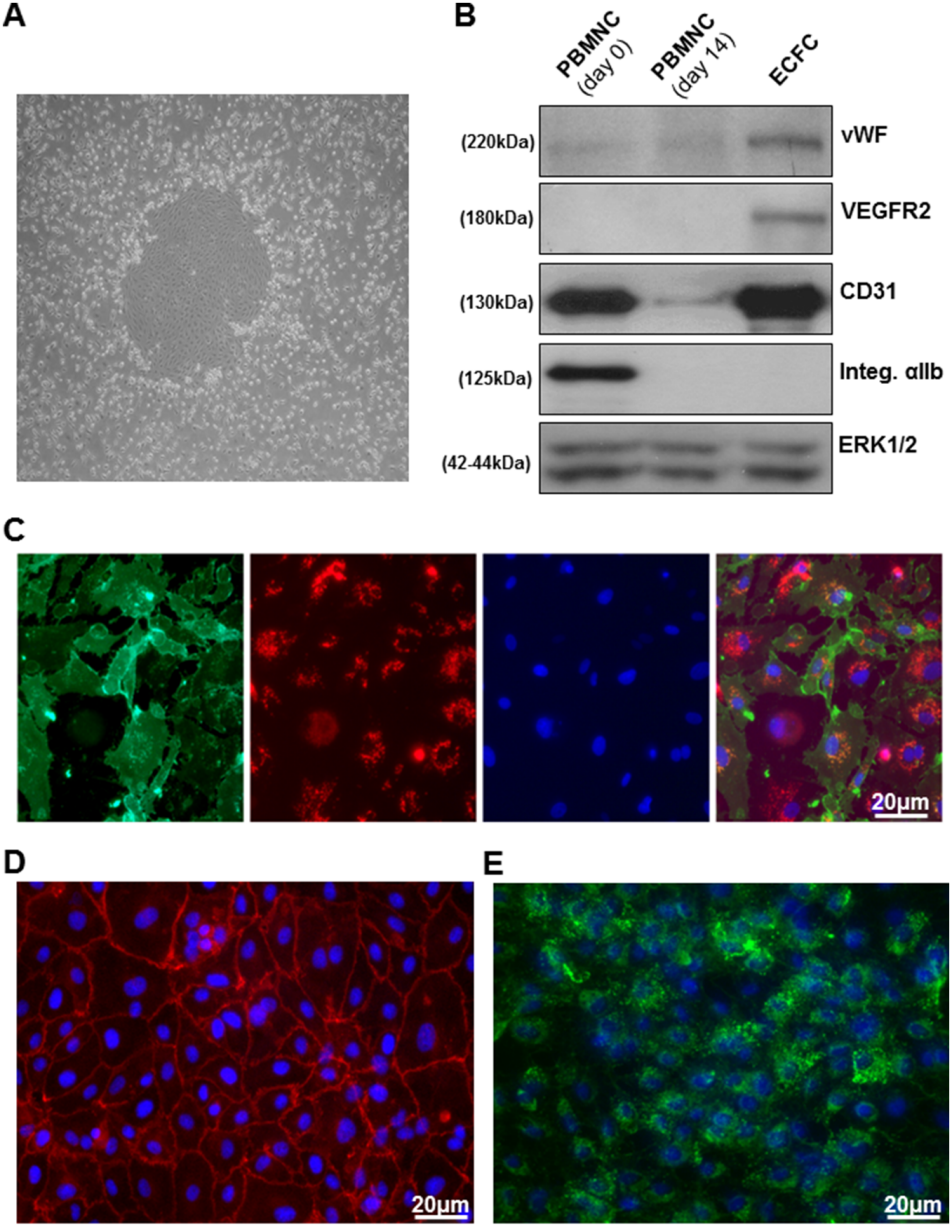
Characterization of the physiological and biochemical phenotype of ECFCs. A typical colony appearing between day 14 and 21 of culture is presented in (A). Immunoblotting for the endothelial markers von Willebrand Factor (VWF), VEGFR2 and CD31, for the platelet marker integrin αIIb and for extracellular signal-regulated kinases 1 and 2 are shown in (B) (from top to bottom). ECFC lysates are compared to lysates from PBMNCs at the time of isolation (day 0) and 14 days after isolation (day 14). In (C), ECFCs were pre-incubated with 10 µg/ml DiI-Ac-LDL (4 hours, 37°C, 5%CO_2_) before staining with 40 µg/ml FITC-UEA and 1 µg/ml 4′,6-diamidino-2-phenylindole (DAPI). Images were taken by confocal immunofluorescence using a Zeiss 510 LSM confocal microscope equipped with a 40X oil-immersion lens. The results displayed are representative of three independent experiments and show the independent channels (FITC, DiI and DAPI from left to right) and the superimposed picture (far right). In (D) and (E), expression and localization of VE-cadherin and VWF were assessed by immunostaining using specific antibodies combined with Alexa Fluor 488 Rabbit Anti-Mouse (green) and Alexa Fluor 546 Donkey Anti-Goat IgG (red) secondary antibodies (1∶200). 4′,6-diamidino-2-phenylindole (blue) (1∶100) was used to localize cell nuclei. The results are representative of three independent experiments.

ECFC colonies were obtained as described in the methods section and an example of a colony is presented in Figure 1A. The development of the endothelial phenotype was assessed by immunoblotting for the endothelial markers von Willebrand Factor (VWF), VEGFR2 and CD31 (Figure 1B). All three markers appeared significantly expressed in ECFCs. While the expression of VWF and VEGFR2 is significant only in the ECFC population, CD31 was also significantly expressed in the initial population of hPBMNC. Interestingly, a significant amount of the platelet-specific marker integrin αIIb in the initial population of hPBMNCs might indicate presence of platelets in the first stages of the culture, which has been previously reported [23]. The endothelial phenotype of the ECFCs obtained in our experiments was further confirmed by testing the ability to take up 1,1′-dioctadecyl–3,3,3′,3′-tetramethyl-indocarbocya-nine-labelled acetylated low-density lipoprotein (DiI-Ac-LDL) and to be stained by fluorescein isothiocyanate-labelled *Ulex europaeus* agglutinin (FITC-UEA) (Figure 1C). The ECFC cultures were also immunostained for VE-cadherin (which resulted in a uniform staining of the cell-cell contacts) and VWF (which highlighted a punctate distribution compatible with the staining of the Weibel-Palade bodies), as shown in Figure 1D and 1E respectively.

**Figure 2:**
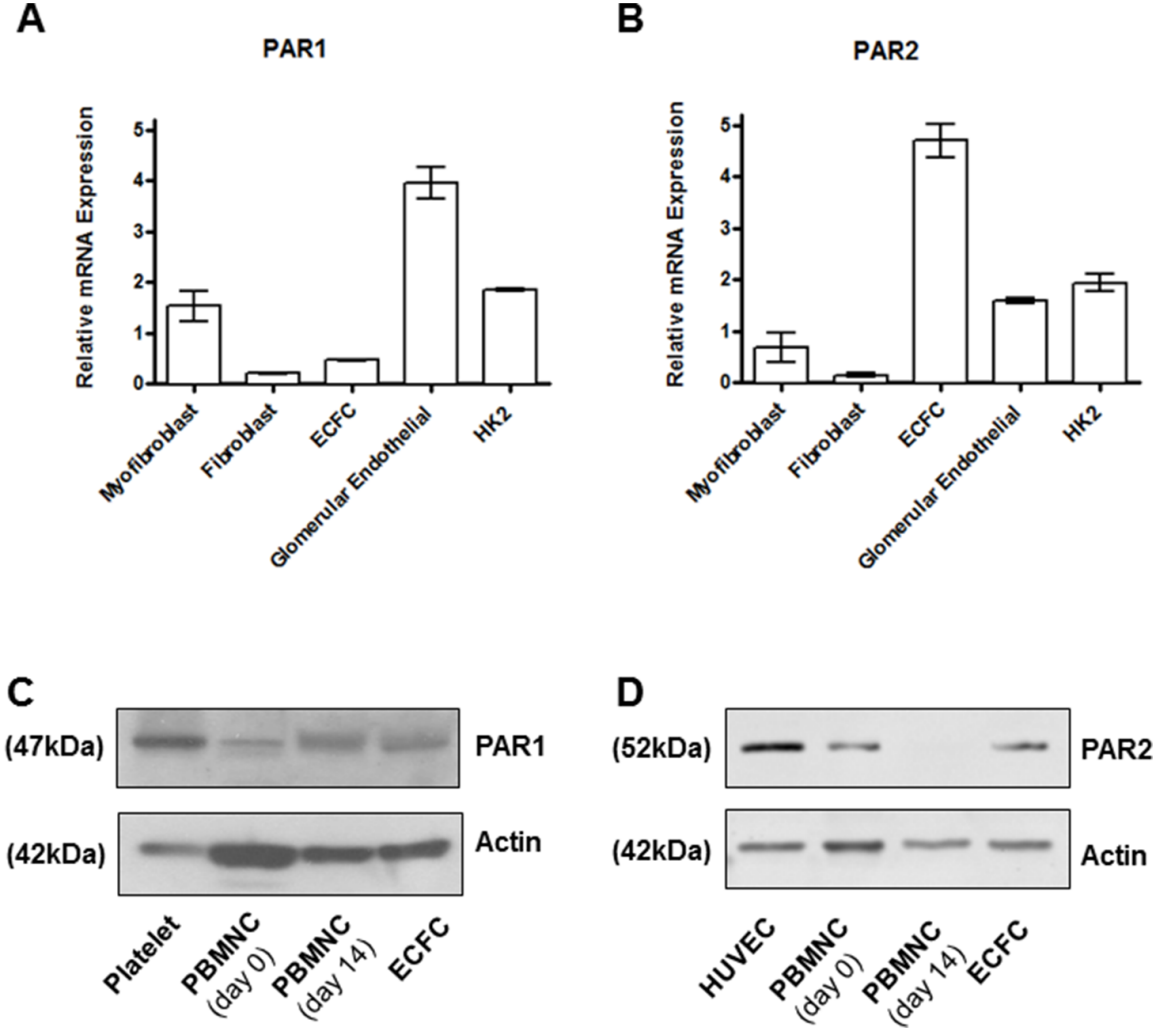
ECFCs express PAR1 and PAR2. (A and B) RNA extracts from fibroblasts, myofibroblasts, ECFCs, glomerular endothelial cells, and epithelial HK2 cells PAR1 and PAR2 expression were analyzed by qPCR using specific primers as described in the Methods section. Relative expressions were calculated according to the 2^−ΔΔCt^ method and normalized to GAPDH. These data are expressed as mean ± standard error of the means (SEM) from three independent experiments. The expression of PAR1 (C) and PAR2 (D) was also examined in platelets or HUVECS (for PAR1 and PAR2 respectively), PBMNCs at days 0 and 14 and in ECFCs (from left to right) by immnunoblotting using selective antibodies (N19). An actin-specific antibody was also utilized to detect the expression of a housekeeping gene in the different lysates. Notably the cells analyzed here display significantly different phenotypes and the actin levels appear different despite equal protein loading for different lanes. The results are representative of three independent experiments.

**Figure 3:**
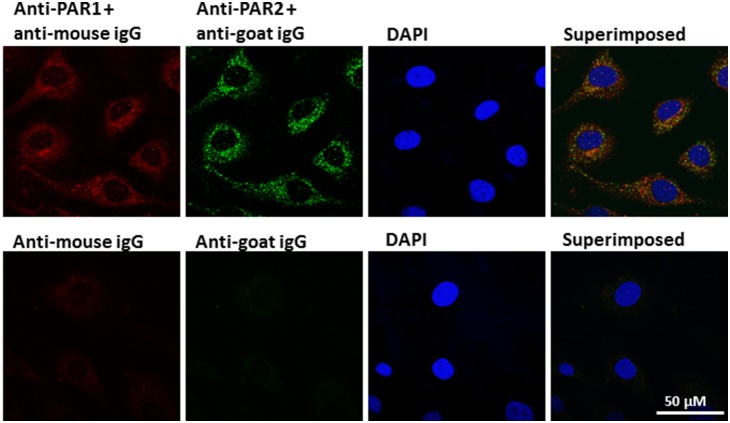
Immunolocalisation of PAR1 and PAR2. ECFCs were cultured to 60–70% confluence and fixed with methanol as described in Methods. Expression and localization of PAR1 and PAR2 were assessed by immunostaining using specific antibodies combined with Alexa Fluor 488 Rabbit Anti-Mouse (green) and Alexa Fluor 546 Donkey Anti-Goat IgG (red) secondary antibodies (1∶200). 4′,6-diamidino-2-phenylindole (blue) (1∶100) was used to localize cell nuclei. The superimposition of the three channels is shown in the far rightpanels, with yellow indicating co-localizing staining for the two receptors. The images are representative of three independent experiments.

The expression of PAR1, PAR2, PAR3 and PAR4 mRNAs was then investigated by RT-PCR in PBMNCs and ECFCs. Only PAR1 and PAR2 were expressed (Figure S1A), while PAR3 and PAR4 were not (data not shown). The absence of PAR4 expression in PBMNCs and ECFCs was confirmed by immunoblotting (Figure S1B), while PAR1 and PAR2 expression was confirmed by qPCR using a different set of specific primers and compared in different cell types (fibroblasts, myofibroblasts, ECFCs and HK2 cells) (Figure 2A and Figure 2B). The expression of PAR1 and PAR2 was confirmed by immunoblotting in both PBMNCs and ECFCs using N-19 antibodies (Figure 2C and Figure 2D, respectively), which have been shown to detect the expression of these receptors in native primary cell types [24]. Platelet and human umbilical vein endothelial cell (HUVEC) protein extracts were used as positive controls. PAR1/2 expression and localization were finally analyzed by immunofluorescence (Figure 3), which confirmed that both receptors are expressed in ECFCs and show a minimal degree of co-localization.

**Figure 4:**
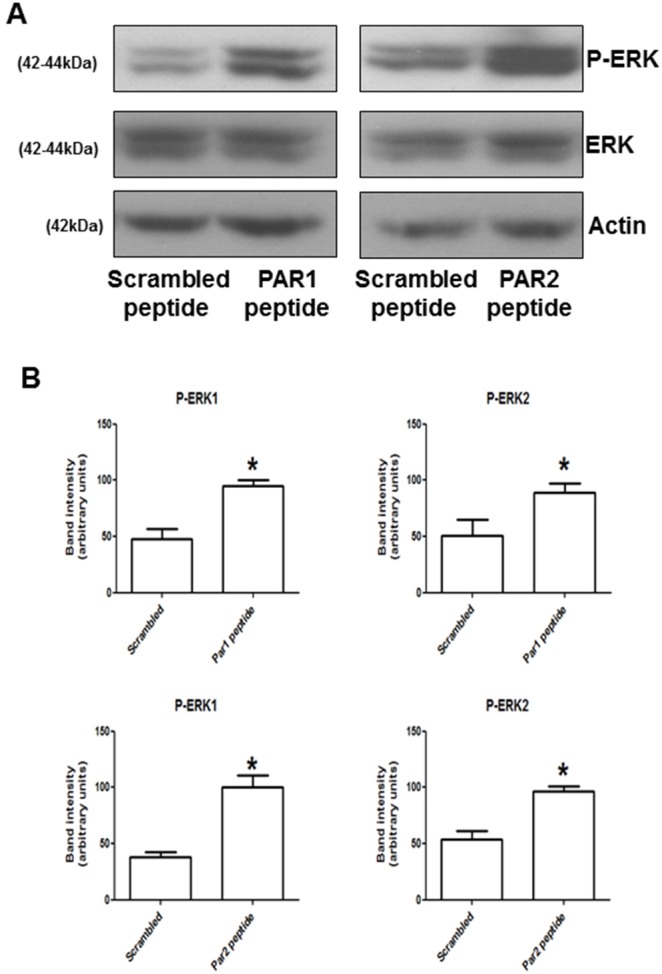
ERK activation by PAR1 and PAR2 stimulation. Proteins were isolated as described in the Methods section from ECFCs after treatment with 50 µM PAR1- (A), or 50 µM PAR2-activating peptide (B) for 30 minutes (37°C, 5% CO_2_). A peptide with a scrambled sequence was utilized at a concentration of 50 µM as a negative control. Phospho-ERK1/2, total ERK1/2 and actin were detected by SDS-PAGE and immunoblotting (from top to bottom). The results are representative of three independent experiments. In (B), phospho-immunoblots were scanned to obtain a digital image and band intensity was analyzed using ImageJ 1.46r. The graphs display the results as arbitrary intensity units for the phospho-ERK1 and phospho-ERK2 bands from 4 independent experiments. Treatment with 50 µM PAR1- or PAR2- activating peptides is compared to treatment with the same concentration of scrambled peptides in A–B and C–D, respectively. The data are mean ± standard error of the means (SEM) and the statistical significance of the difference was tested by t-test for paired samples (* = p<0.05).

**Figure 5:**
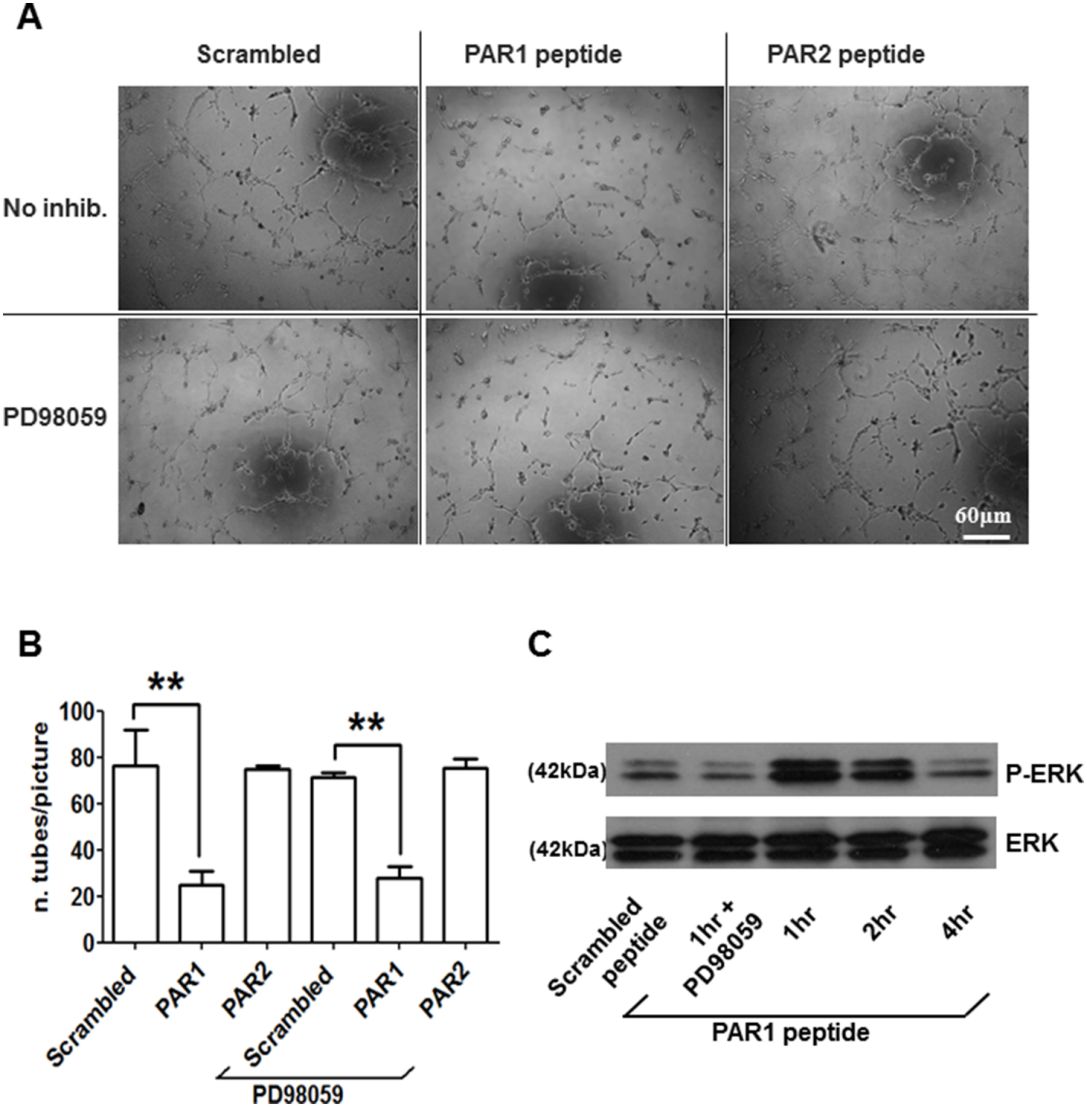
The activation of PAR1 inhibits capillary-like tube formation by ECFCs on Matrigel. 10^4^ ECFCs/well were plated onto Matrigel matrix in tube formation assay buffer (i.e. EBM-2 medium containing 2% FBS). Cells were cultured with 50 µM scrambled control peptide, 50 µM PAR1-activating peptide or 50 µM PAR2-activating peptide in the absence or presence of 50 µM PD98059 (as indicated). Phase contrast images were captured 4 hours after cell seeding. Representative examples from 4 independent experiments are shown in (A). The number of tubes per image was quantified using the Angiogenesis Analyzer plugin for ImageJ. Means ± SEM from three independent experiments are shown in (B). Statistical significance was tested by one-way ANOVA with Bonferroni post-test (** = p<0.05). Time-dependent activation of ERK1/2 by PAR1-activating peptide (0, 1, 2 and 4 hours) and its inhibition by 50 µM PD98059 were examined using a phospho-specific ERK1/2 antibody and immunoblotting (C). The results are representative of three independent experiments.

**Figure 6:**
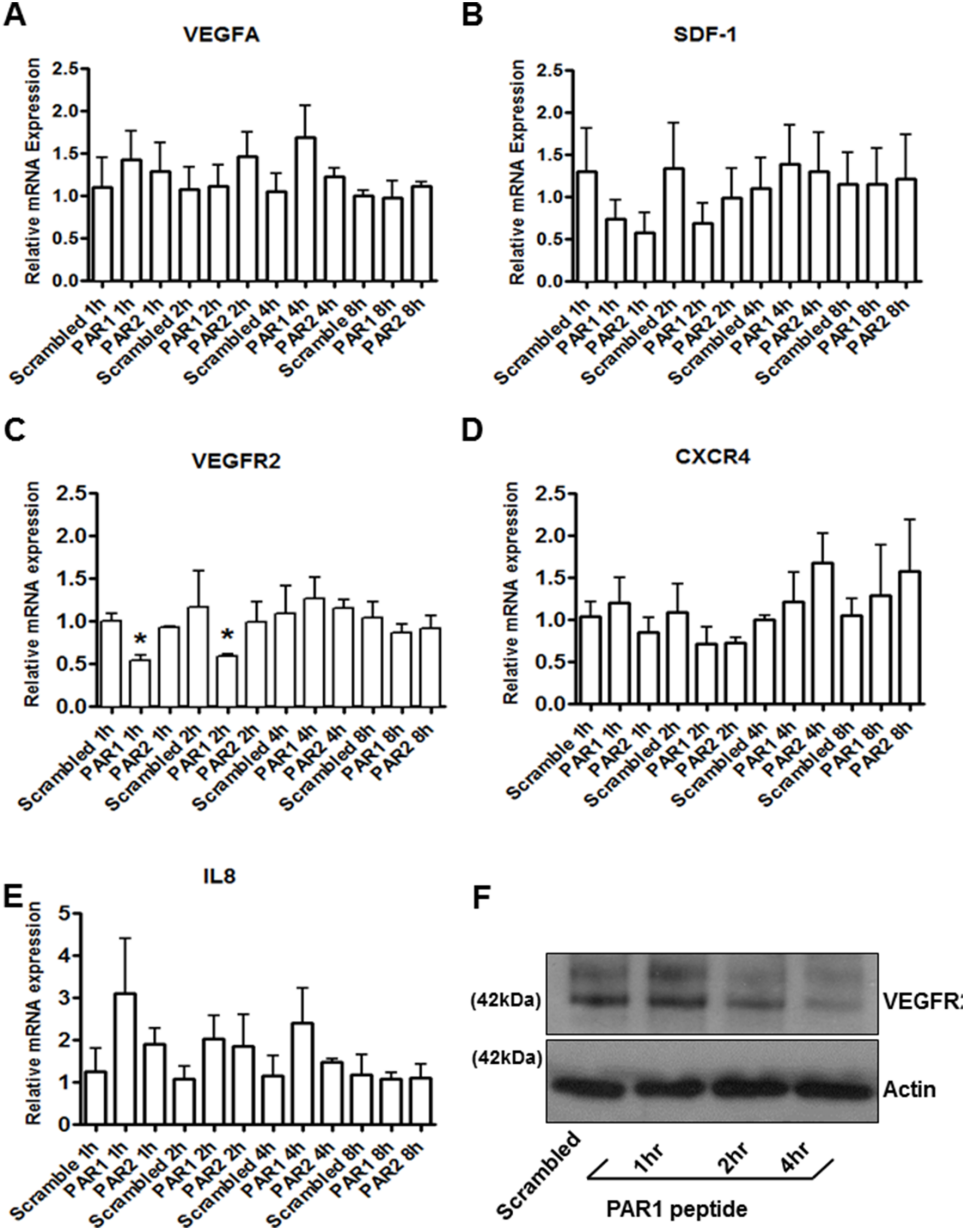
Downregulation of VEGFR2 by PAR1 stimulation. (A–E) Time course of relative expression of pro-angiogenic marker genes following PARs-stimulation. Total RNA was extracted at 1, 2, 4 and 8 hours after stimulation and relative expressions were calculated according to the 2^−ΔΔCt^ method and normalized to GAPDH. These data are expressed as mean ± standard error of the means (SEM) from three independent experiments (* = p<0.05, compared to scrambled peptide treatment at matched time point). (F) Proteins were isolated as described in the Methods section from ECFCs after treatment with 50 µM PAR1-activating peptide for 0, 1, 2 and 4 hours. VEGFR2 and actin were detected by SDS-PAGE and immunoblotting. The results are representative of three independent experiments.

Next, we investigated whether PAR1 and PAR2 are functionally coupled to the extracellular signal-regulated kinase pathway (ERK1/2). A specific antibody for phosphorylated ERK1/2 was utilized to assess ERK activation. In these experiments, PAR1- or PAR2-stimulation (Figure 4A) resulted in a significant increase in ERK1/2 activation. The phosphorylation levels of ERK1 and ERK2 were quantified by densitometry and showed significant activation by both PAR1 and PAR2 activating peptides (Figure 4B). Next, the effect of PAR1 and PAR2 stimulation on the tubulogenic activity of ECFCs was investigated *in vitro* by monitoring capillary-like tube formation on Matrigel. Stimulation of PAR1, but not PAR2 or treatment with scrambled peptide, inhibited tube formation by ECFCs in this assay (Figure 5A–B). Interestingly, treatment with 50 µM PD98059 did not affect basal tube formation by ECFCs and did not interfere with PAR1-dependent inhibition of tubulogenesis, suggesting that ERK activation is not primarily involved in tube formation or responsible for its inhibition by PAR1 agonist peptide. These capillary tube formation data were re-analysed using “total tube length” as readout without changing the interpretation of the data (Figure S3A). The efficient inhibition of ERK1/2 phosphorylation in the presence of 50 µM PD98059 was confirmed by immunoblotting for phospho-ERK1/2 (Figure 5C). In view of the inhibitory effect of PAR1 activation on tubulogenesis, we examined the expression of the vasculogenic factors VEGF-A and SDF-1 and their receptors VEGFR2 and CXCR4 in the presence of PAR1-activating peptide [25]–[27] (Figure 6A–E). We utilized qPCR and showed a significant decrease in VEGFR2 mRNA levels after 1 and 2 hours of treatment with PAR1-activating peptide, while at longer incubations VEGFR2 mRNA levels returned to control levels (Figure 6A). Other vasculogenic genes showed some changes in response to PAR1 stimulation, but the changes did not reach statistical significance (Figure 6B–E). The down-regulation of VEGFR2 by PAR1-activating peptide was confirmed at the protein level and was evident after incubation times of 2 and 4 hours, but not 1 hour (Figure 6F). Densitometric analysis of VEGFR2 immunoblots is shown in Figure S4.

**Figure 7:**
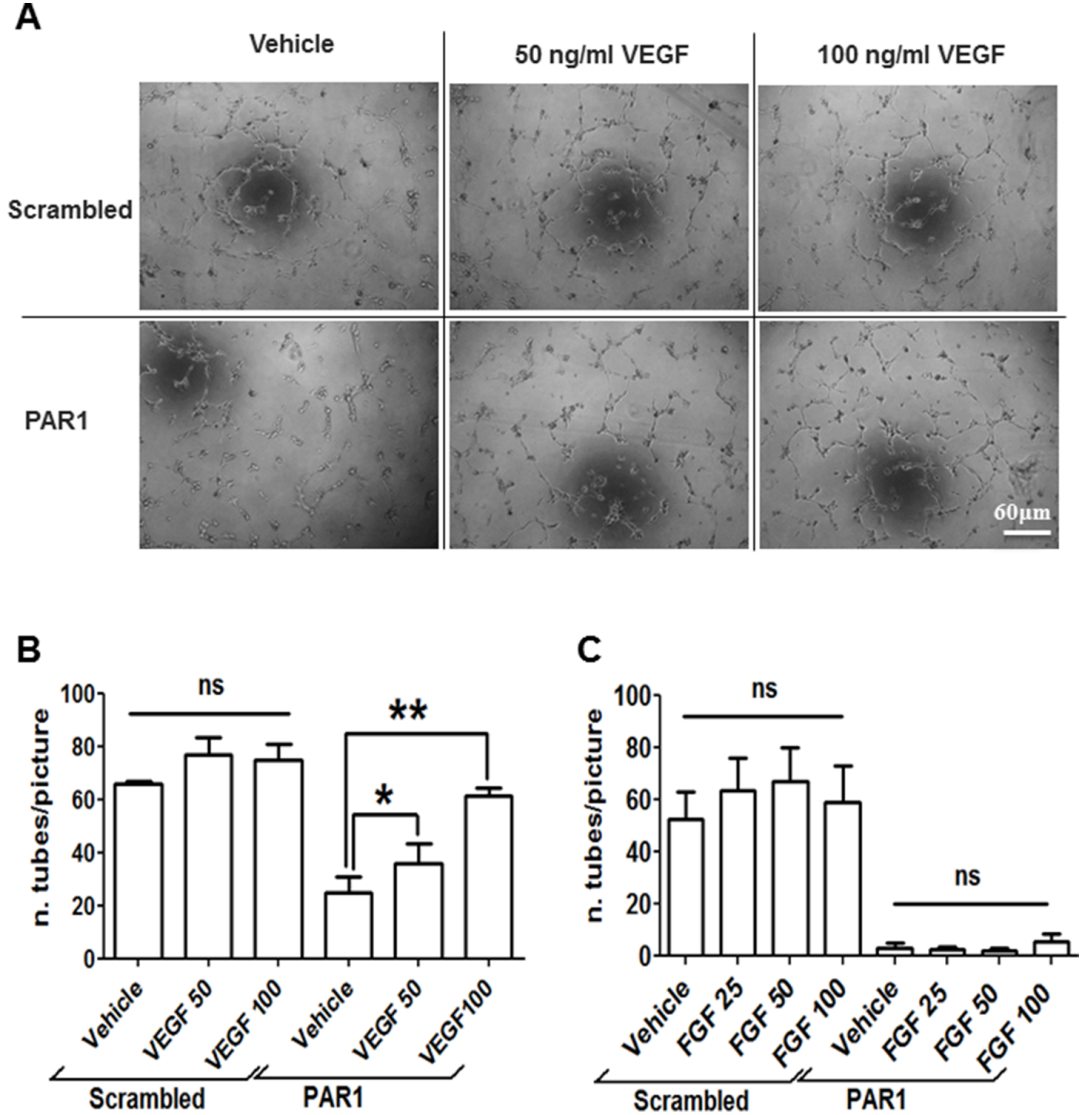
The inhibition of capillary-like tube formation by PAR1-activating peptide can be reversed by increasing concentrations of VEGF. 10^4^ ECFCs/well were plated onto Matrigel matrix in tube formation assay buffer (i.e. EBM-2 medium containing 2% FBS). Cells were cultured with 50 µM scrambled control peptide or 50 µM PAR1-activating peptide in the absence or presence VEGF (50 and 100 ng/ml) (A and B) or FGF (25, 50 and 100 ng/ml) (C). 4 hours after seeding, images were collected and representative examples of three independent experiments are shown in (A). The number of tubes per image was calculated using the Angiogenesis Analyzer plugin for ImageJ. Means ± SEM from three independent experiments are shown in (B) and (C). Statistical significance was tested by one-way ANOVA with Bonferroni post-test (* = p<0.05; ** = p<0.01).

Finally, in order to confirm that the anti-tubulogenic effect of PAR1 is mediated by down-regulation of VEGFR2 and reduced responsiveness to VEGF in the Matrigel, we attempted to rescue the formation of capillary-like tubes by addition of exogenous VEGF to ECFC treated with PAR1-activating peptide. As shown in Figure 7, the addition of 50 or 100 ng/ml VEGF reversed the inhibition of tube formation induced by PAR1-activating peptide. The re-analysis of these data using “total tube length” as readout is in Figure S3B, which also shows significant rescue of capillary tube formation by both 50 and 100 ng/ml VEGF. On the other hand, basic Fibroblast Growth Factor (FGF) did not rescue tube formation in the presence of PAR1-activating peptide at any of the tested concentrations (25, 50 and 100 ng/ml, Figure 7C and Figure S3C). Interestingly, neither VEGF (50 or 100 ng/ml) nor FGF (25, 50 or 100 ng/ml) significantly increased capillary tube formation in the absence of PAR1-activating peptide.

## Discussion

Mature endothelial cells have been shown to express PAR1, PAR2, PAR3 and PAR4 [28]. PAR1 has been shown to play an important role in endothelial responses to thrombin, such as junctional remodeling, vascular permeability regulation, paracrine activity, migration [29], inflammatory responses in endothelial cells [30], [31] and vascular relaxation [32]. PAR2 has been implicated in the regulation of inflammatory responses of endothelial cells, including the development of a pro-thrombotic endothelial state and the upregulation of cyclooxygenases [30], [31], [33]. PAR4 has been shown to play some roles in pro-inflammatory responses in endothelial cells but its major roles remain elusive [33], [34].

On the other hand, previous studies have only reported on the expression of PAR1 in different EPC types. Bone marrow-derived early EPC [18], cord blood CD34^+^-derived EPC [17], and both cord and adult peripheral blood late EPCs [19] have only been shown to express PAR1. In this study we present evidence of expression of PAR1 in adult peripheral blood ECFCs and further extend these studies by demonstrating the expression of PAR2, but not PAR3 or PAR4 in these cells. In previous studies, PAR1 activation has been associated with cell differentiation, upregulation of endothelial markers in EPCs (VE-cadherin, [18]) and increased expression of pro-angiogenic factors or their receptors (stromal cell-derived factor 1/CXCR4 [17] and interleukin-8 [19]). In our qPCR experiments, we observed a trend towards increased expression of CXCR4 and IL8 in response to PAR1 activation (Figure 6D and 6E); however, we failed to find a statistically significant change in the expression of these markers. In the case of CXCR4, this discrepancy might result from the difference in the time points analyzed in our and the previous study (i.e. 1–8 hours in our study vs 24 hours in previous studies). The upregulation of pro-angiogenic markers in response to PAR1 stimulation observed in previous studies was accompanied by a pro-angiogenic response *in vitro*, in particular capillary-like tube formation [17], [19]. A previous report by Smadja and colleagues showed that PAR1 stimulation induced capillary-like tube formation by EPCs by up-regulating CXCR4 and potentiating signalling via the stromal cell-derived factor 1α (SDF-1α)/CXCR4 axis [17]. In disagreement with this study, we observed that PAR1 activation decreased tubulogenesis by ECFCs below basal levels. The difference in the starting cell population in the isolation protocol is, again, the most likely explanation for this discrepancy (i.e. adult peripheral blood mononuclear cells without cell sorting in our study versus cord blood cells with cd34+ cell sorting in the above study [17]). Other minor differences in the culture procedures are: 1) different fetal bovine serum concentration in the culture medium (12% in our study versus 2–5% in the other studies [17]–[19]); and 2) extracellular matrix coating of the culture vessels (collagen in our study versus gelatin [17], [19] or fibronectin [18] in the other studies). The protocol that we utilized was previously described in one of the most influential papers in the field [2]. The ECFCs used in our study are characterized by a stronger vasculogenic response compared to EPCs from the above study, as suggested by significant tube formation at earlier time points (4 hours culture on Matrigel for maximal response in our study *versus* measurements after 18 hours on Matrigel in the above study [17]). Previous studies suggested that upregulation of CXCR4 and subsequent increased signaling through the CXCR4/SDF-1α axis is responsible for tubulogenesis in response to PAR1 activation [17]. In this study, we investigated the expression of CXCR4 and SDF-1 and did not detect significant modulation by PAR1/2 stimulation. Some controversy regarding the role of PAR1 in the regulation of capillary-like tube formation by mature endothelial cell types also exists. Despite a general agreement that PAR1 activation is a pro-angiogenic stimulus in mature endothelial cells, PAR1 activation by thrombin has also been shown to inhibit tube formation on Matrigel by HUVECs [35]. Moreover, PAR1 activation by thrombin in human microvascular endothelial cells displayed dual effects on tube formation on Matrigel, with low concentrations stimulating and high concentrations inhibiting tubulogenesis [36]. Data presented in this study suggest that VEGFR2 is down regulated by PAR1 activation in ECFCs. The importance of this receptor for vasculogenic responses of EPCs has been reported previously [37]. The relevance of VEGFR2 downregulation for PAR1-dependent inhibition of tubulogenesis was confirmed by the rescue of normal capillary-like tube formation in the presence of both PAR1-activating peptides and high concentrations of exogenous VEGF (50 or 100 ng/ml). This suggests that PAR1 activation decreases VEGF signalling by reducing VEGFR2 density on the surface of ECFCs and that high concentrations of exogenous VEGF are necessary to restore sufficient levels of VEGF-dependent signalling and guarantee tube formation. Interestingly, VEGF on its own does not increase tube formation (which suggests that in the absence of PAR1 activation and VEGFR2 downregulation the concentration of VEGF in Matrigel is sufficient to achieve full response) and addition of equally high concentrations of FGF (25, 50 or 100 ng/ml) does not rescue the inhibition of capillary tube formation induced by PAR1 activation (which suggests that the restoration of sufficient levels of VEGF signalling is necessary for the tubulogenic response to occur in the presence of PAR1-activating peptide).

It seems important to highlight here that the PAR1 stimulation has previously been shown to accelerate the proliferation of endothelial progenitor cells similar to the ones described in this study [17], [19]. Our observation of an inhibition of the tubulogenic response of ECFCs by PAR1 activation is not in contrast with these previous reports. In fact, the tubulogenic response analyzed here is unlikely to be affected by cell proliferation due to its short time course (i.e. 4 hours are not sufficient for a significant effect of cell proliferation on cell number and tube formation). The phenomena analyzed in our vasculogenic assay are related to cell migration, cytoskeletal rearrangement and establishment of cell-cell contacts.

Although PAR2 shares some aspects of its signal transduction with PAR1, such as activation of the MEK-ERK pathway [17], [28], [38], these two receptors affect ECFC tubulogenic responses differently. In our study, although both PAR1 and PAR2 coupled to ERK signaling pathways, only PAR1 activation inhibited ECFC tube formation. This, together with the lack of effect of the inhibitor PD98959, suggests that ERKs do not play a significant role in the regulation of capillary-like tube formation by ECFCs.

Taken together, we show that PAR1 and PAR2 are both expressed in ECFCs. These receptors are functionally coupled to the ERK1/2 signaling pathway, but only PAR1 stimulation leads to VEGFR2 down-regulation and inhibition of tubulogenesis. These observations can be utilized to modulate the vasculogenic activity of ECFCs for cell therapy and tissue engineering purposes.

## Supporting Information

Figure S1 **PAR1 and PAR2 expression in ECFCs.**
(PDF)

Figure S2 **VEGF-dependent stimulation of capillary-like tube formation by HUVECs.**
(PDF)

Figure S3 **Re-analysis of tube formation experiments shown in** **Figures 5** **and** **7** **using “total tube length” instead of “tube number”.**
(PDF)

Figure S4 **Densitometric analysis of VEGFR2 immunoblots.**
(PDF)
