# Supplementary material for: Expression of Protease-Activated Receptor 1 and 2 and Anti-Tubulogenic Activity of Protease-Activated Receptor 1 in Human Endothelial Colony-Forming Cells
Source: PLoS One. 2014 Oct 7;9(10):e109375. doi: 10.1371/journal.pone.0109375 (PMC4188577; doi:10.1371/journal.pone.0109375)
Supplement: Figure S1 — PAR1 and PAR2 expression in ECFCs. (PDF) [file pone.0109375.s001.pdf]

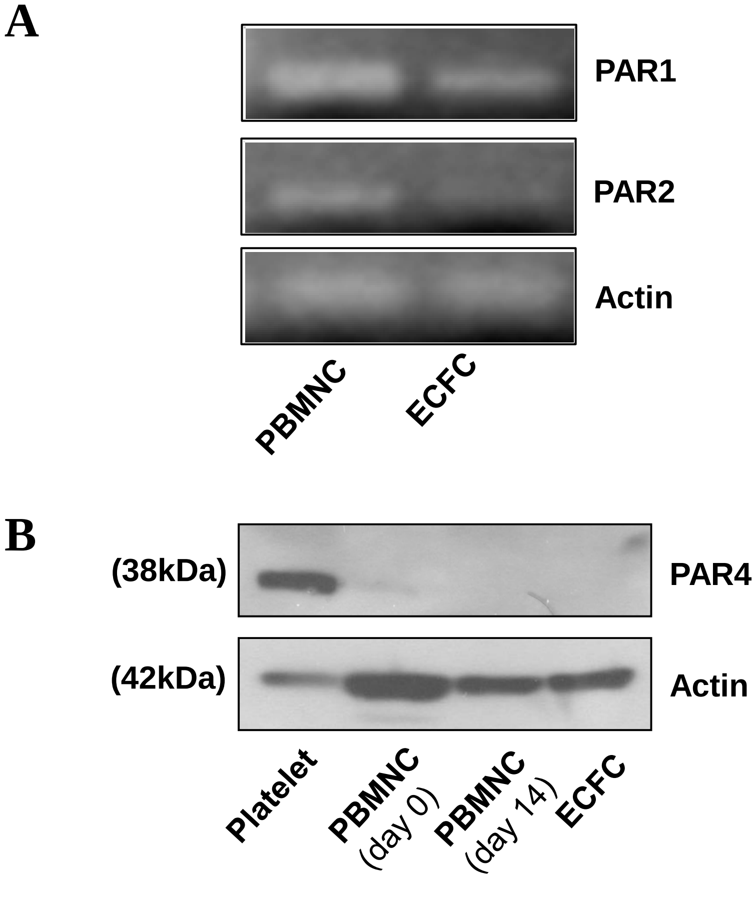

Supplementary figure 1: PAR1 and PAR2 expression in ECFCs. (A) The expression of PAR1, PAR2 and actin has been detected in PBMNCs and ECFCs by RT-PCR as described in the methods section. **(B)** The expression of PAR4 has been investigated by immunoblot in platelets, PBMNCs at day 0, in PBMNCs at day 14 and in ECFCs (from left to right). Actin expression was utilized as a loading control.
