# Supplementary material for: Expression of Protease-Activated Receptor 1 and 2 and Anti-Tubulogenic Activity of Protease-Activated Receptor 1 in Human Endothelial Colony-Forming Cells
Source: PLoS One. 2014 Oct 7;9(10):e109375. doi: 10.1371/journal.pone.0109375 (PMC4188577; doi:10.1371/journal.pone.0109375)
Supplement: Figure S2 — VEGF-dependent stimulation of capillary-like tube formation by HUVECs. (PDF) [file pone.0109375.s002.pdf]

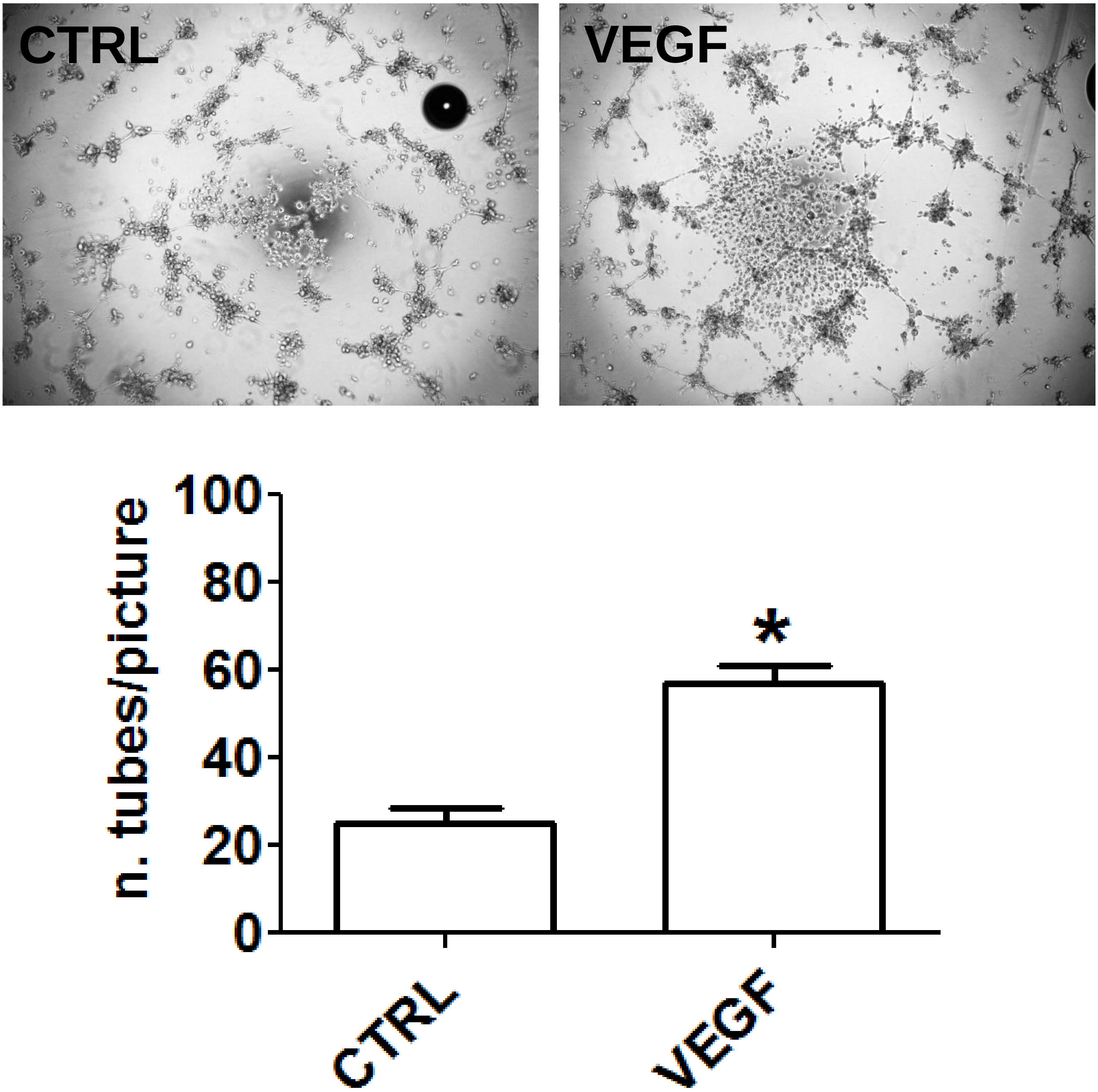

Supplementary Figure 4. VEGF-dependent stimulation of capillary-like tube formation by HUVECs. 10^4 HUVECs/well were cultured in growth factor-reduced medium were plated onto Matrigel™ matrix and incubated for 8 hours with or without 50 ng/ml VEGF. Representative images from 4 independent experiments are shown in (**A**). The number of tubes per image was quantified using the Angiogenesis Analyzer plugin for ImageJ. Means ± SEM from three independent experiments are shown in (**B**). Statistical significance was tested by one-way ANOVA with Bonferroni post-test (* = $p<0.05$).
