# Supplementary material for: Expression of Protease-Activated Receptor 1 and 2 and Anti-Tubulogenic Activity of Protease-Activated Receptor 1 in Human Endothelial Colony-Forming Cells
Source: PLoS One. 2014 Oct 7;9(10):e109375. doi: 10.1371/journal.pone.0109375 (PMC4188577; doi:10.1371/journal.pone.0109375)
Supplement: Figure S3 — Re-analysis of tube formation experiments shown in Figures 5 and 7 using “total tube length” instead of “tube number”. (PDF) [file pone.0109375.s003.pdf]

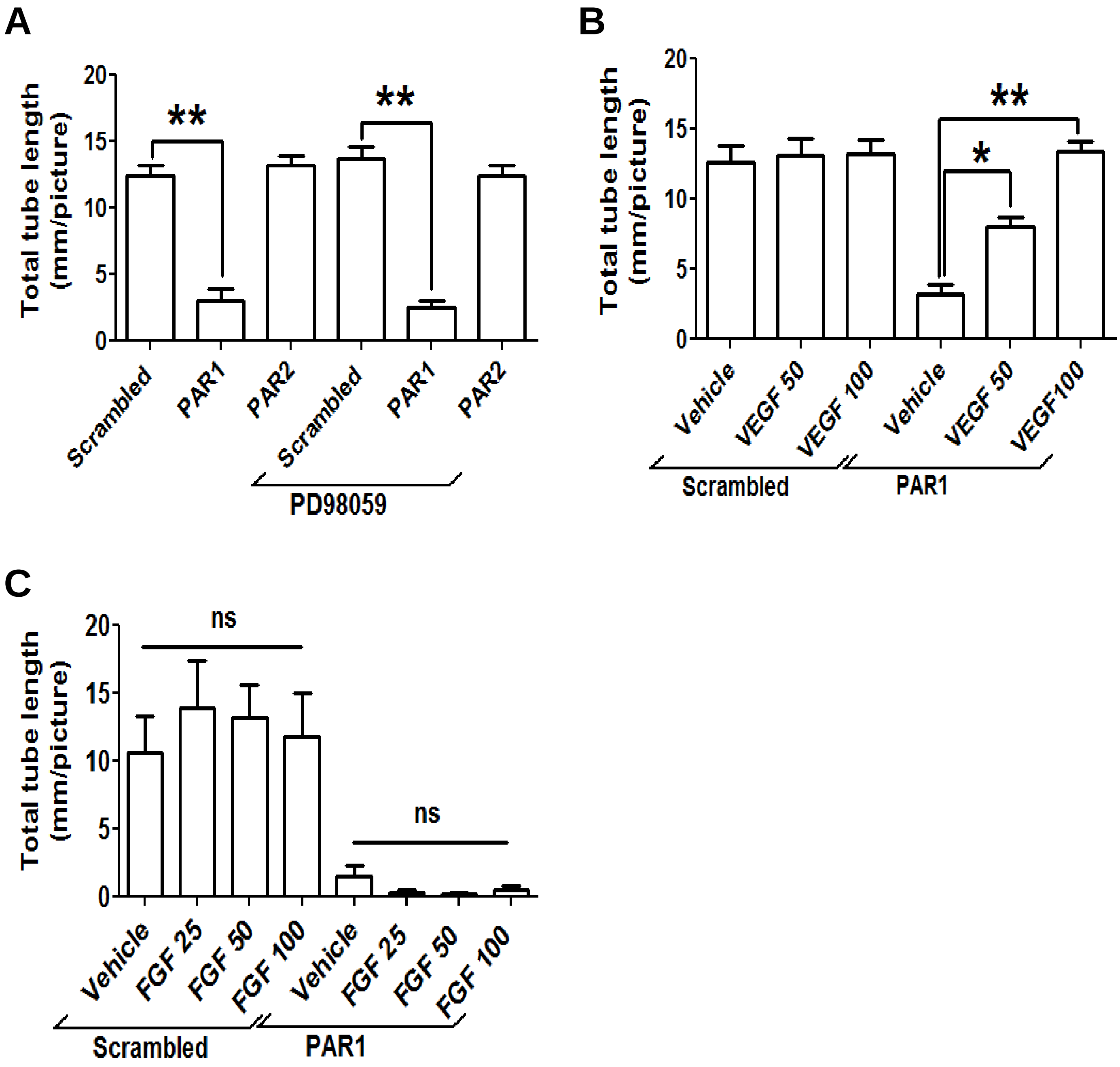

Supplementary figure 3: Re-analysis of tube formation experiments shown in Figures 5 and 7 using "total tube length" instead of "tube number". 10^4 ECFCs/well were plated onto Matrigel™ matrix in EBM-2 medium + 2% FBS. In (A), cells were cultured with 50 µM scrambled control peptide, 50 µM PAR1-activating peptide or 50 µM PAR2-activating peptide in the absence or presence of 50 µM PD98059 (as indicated). In (B), cells were cultured with 50 µM scrambled control peptide or 50 µM PAR1-activating peptide in the absence or presence VEGF (50 and 100 ng/ml). In (C), cells were cultured with 50 µM scrambled control peptide or 50 µM PAR1-activating peptide in the absence or presence FGF (25, 50 and 100 ng/ml). 4 hours after seeding, images were collected and representative examples of three independent experiments are shown in (A). The total tube length per image was calculated using the Angiogenesis Analyzer plugin for ImageJ and expressed in mm/picture. Means ± SEM from three independent experiments are shown in (B). Statistical significance was tested by one-way ANOVA with Bonferroni post-test (** = $p<0.01$, * = $p<0.05$).
