# Supplementary material for: Expression of Protease-Activated Receptor 1 and 2 and Anti-Tubulogenic Activity of Protease-Activated Receptor 1 in Human Endothelial Colony-Forming Cells
Source: PLoS One. 2014 Oct 7;9(10):e109375. doi: 10.1371/journal.pone.0109375 (PMC4188577; doi:10.1371/journal.pone.0109375)
Supplement: Figure S4 — Densitometric analysis of VEGFR2 immunoblots. (PDF) [file pone.0109375.s004.pdf]

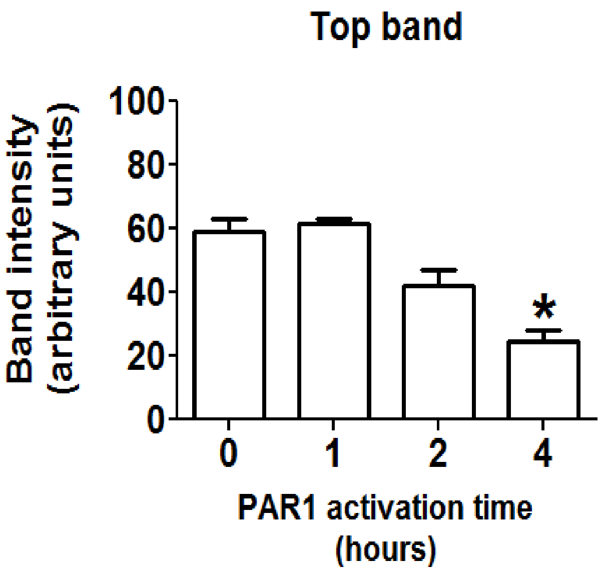

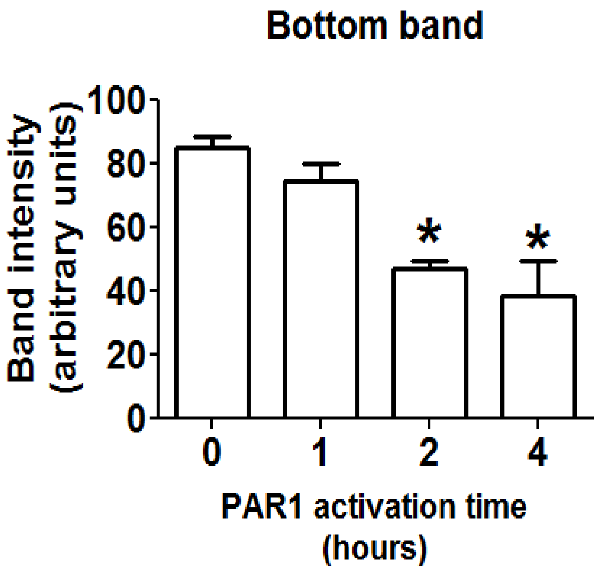

Supplementary figure 4: Densitometric analysis of VEGFR2 immunoblots. VEGFR2 expression has been investigated by immunoblotting using a specific antibody as described in the Methods section and shown in Figure 6F. Immunoblots were scanned to obtain a digital image and band intensity was analyzed using ImageJ 1.46r. The graphs display the results as arbitrary intensity units for the VEGFR2 at 4 time points from 3 independent experiments. The receptor appears in immunoblots as a dimer, as described previously. Here we show separate densitometric analysis of the two bands. The data are mean ± standard error of the means (SEM) and the statistical significance of the difference was tested by one-way ANOVA with Bonferroni post-test (* = $p<0.05$ compared to no treatment, i.e. 0 hours).
